# Hemin binding by *Porphyromonas gingivalis* strains is dependent on the presence of A‐LPS

**DOI:** 10.1111/omi.12178

**Published:** 2017-03-09

**Authors:** M. Rangarajan, J. Aduse‐Opoku, N.A. Paramonov, A. Hashim, M.A. Curtis

**Affiliations:** ^1^ Institute of Dentistry Barts and The London School of Medicine & Dentistry Queen Mary University of London London UK; ^2^ College of Dentistry King Faisal University Al‐Ahsa Saudi Arabia

**Keywords:** A‐LPS, hemin binding, lipopolysaccharides, pigmentation, *Porpyhromonas gingivalis*

## Abstract

*Porphyromonas gingivalis* is a Gram‐negative black pigmenting anaerobe that is unable to synthesize heme [Fe(II)‐protoporphyrin IX] or hemin [Fe(III)‐protoporphyrin IX‐Cl], which are important growth/virulence factors, and must therefore derive them from the host. *Porphyromonas gingivalis* expresses several proteinaceous hemin‐binding sites, which are important in the binding/transport of heme/hemin from the host. It also synthesizes several virulence factors, namely cysteine‐proteases Arg‐ and Lys‐gingipains and two lipopolysaccharides (LPS), O‐LPS and A‐LPS. The gingipains are required for the production of the black pigment, μ‐oxo‐bisheme {[Fe(III)PPIX]_2_ O}, which is derived from hemoglobin and deposited on the bacterial cell‐surface leading to the characteristic black colonies when grown on blood agar. In this study we investigated the role of LPS in the deposition of μ‐oxo‐bisheme on the cell‐surface. A *P. gingivalis* mutant defective in the biosynthesis of Arg‐gingipains, namely *rgpA/rgpB*, produces brown colonies on blood agar and mutants defective in Lys‐gingipain (*kgp*) and LPS biosynthesis namely *porR*,* waaL*,* wzy*, and *pg0129* (α‐1, 3‐mannosyltransferase) produce non‐pigmented colonies. However, only those mutants lacking A‐LPS showed reduced hemin‐binding when cells in suspension were incubated with hemin. Using native, de‐*O*‐phosphorylated and de‐lipidated LPS from *P. gingivalis* W50 and *porR* strains, we demonstrated that hemin‐binding to O‐polysaccharide (PS) and to the lipid A moiety of LPS was reduced compared with hemin‐binding to A‐PS. We conclude that A‐LPS in the outer‐membrane of *P. gingivalis* serves as a scaffold/anchor for the retention of μ‐oxo‐bisheme on the cell surface and pigmentation is dependent on the presence of A‐LPS.

## 1 INTRODUCTION

The black pigmenting anaerobe *Porphyromonas gingivalis* is a major pathogen in chronic adult periodontal disease^1^ and has recently been described as a “keystone pathogen” with wide‐ranging effects critical for the development of dysbiosis and disease progression.^2^ The main habitat of this organism is the human gingival crevice where nutrients are gained from gingival crevicular fluid ‐ a plasma exudate. Hemin [Fe(III)‐protoporphyrin IX‐Cl] is an important requirement for growth of *P. gingivalis* 3, 4 and as the organism is not able to synthesize protoporphyrin IX ring^5^, ^6^ and does not contain any siderophores,^7^, ^8^ the major source of heme [Fe(II)‐protoporphyrin IX] is therefore the host.

Black‐pigmenting *Bacteroides* species have been shown to degrade plasma proteins involved in the transport and conservation of body iron, namely albumin, hemopexin, haptoglobin, and transferrin, to varying degrees, with *Bacteroides gingivalis* (*P. gingivalis*) being the most effective.^9^ The cysteine protease Lys‐gingipain (Kgp) of *P. gingivalis* can cleave free hemoglobin,^10^ haptoglobin, hemopexin, and transferrin in human serum but was not able to degrade hemoglobin, or the β‐chain of haptoglobin when these were present in a haptoglobin‐hemoglobin complex in serum.^11^

*Porphyromonas gingivalis* possesses additional outer membrane proteins that are important in the binding and transport of heme and which form part of the *hmu* hemin‐uptake locus,^12^ namely HmuY,^13^ and HBP35 protein has been described as an important hemin‐binding protein.^14^ Several putative TonB‐dependent outer‐membrane receptors have been described including Tlr,^15^ IhtA (iron heme transport),^16^ HmuR (hemin utilization receptor),^6^, ^17^, ^18^ and HemR (hemin‐regulated receptor).^19^ HmuR exhibited amino acid sequence homology to TonB‐dependent receptors involved in heme, vitamin B12 or iron‐siderophore transport in other bacteria.^17^ A *P. gingivalis hmuR* isogenic mutant strain was shown to have impaired growth on hemin and hemoglobin as sole source of iron and showed decreased ability to bind hemin and hemoglobin. *Escherichia coli* cells overexpressing *P. gingivalis* HmuR as well as purified recombinant HmuR were able to bind hemin, hemoglobin and serum albumin‐hemin complex.^17^

*Porphyromonas gingivalis* W50 produces several virulence factors including gingipain proteases and two lipopolysaccharides (LPSs), namely O‐LPS^20^ and A‐LPS.^21^

In this study, we addressed the question whether the high abundance low‐affinity hemin‐binding site described by Tompkins et al.^22^ may be one of the LPS of *P. gingivalis*. To test this hypothesis, we examined a variety of isogenic mutant strains of *P. gingivalis* lacking Arg‐gingipains, Lys‐gingipain and defective in the biosynthesis of O‐LPS and A‐LPS for their ability to pigment and to bind hemin not only to whole cells but also to LPS, de‐phosphorylated LPS and de‐lipidated LPS. Mutant strains of *P. gingivalis*:* porR* (PG1138), which is defective in A‐LPS synthesis, and *galE* (PG0347), which synthesizes a truncated O‐PS repeating unit of O‐LPS, are described in greater detail here in this manuscript. Shoji et al.^23^ described the *porR* mutant strain in *P. gingivalis* ATCC33277 isolated by transposon and targeted mutagenesis and Gallagher et al.^24^ have referred to a *porR* mutant strain isolated by inactivation of PG1138 in *P. gingivalis* W50.

*Porphyromonas gingivalis galE* (PG0347) shares homology with *galE* of *E. coli*, which encodes UDP‐galactose‐4‐epimerase, responsible for the conversion of UDP‐Glc to UDP‐Gal. Galactose is a component of the repeating unit of O‐PS^20^ and in *galE* (ΔPG0347), O‐LPS is still synthesized, but its repeating unit is shortened by one residue, namely Gal (Unpublished data, N. Paramonov, J. Aduse‐Opoku, M. Rangarajan and M.A. Curtis).

The results of hemin‐binding to the mutant strains of *P. gingivalis* exhibit a consistent pattern, which suggests that the deposition of μ‐oxo‐bisheme on the cell surface of the *P. gingivalis* strains appears to be related to the synthesis/presence of A‐LPS in the outer leaflet of the outer membrane. We propose that the presence of A‐LPS serves as a matrix for the deposition of μ‐oxo‐bisheme on the *P. gingivalis* cell surface.

## 2 MATERIALS AND METHODS

### 2.1 Materials

A solution containing 30% acrylamide‐*N*,*N*‐methylenebisacrylamide (BIS; 37.5:1) was obtained from Bio‐Rad Laboratories (Hercules, CA, USA). Horseradish peroxide‐labeled mouse immunoglobulin was purchased from Dako A/S (High Wycombe, UK). All other chemicals were from VWR (Lutterworth, UK) or from Sigma‐Aldrich Co. Ltd (Poole, UK) and were the purest grades available. *N*α‐acetyl‐Lys‐*p*‐nitroanilide was obtained from Bachem Feinchemikalein AG (Bubendorf, Switzerland). Hemin was obtained from Roche (Burgess Hill, UK). Restriction and modification enzymes were purchased from New England BioLabs (Ipswich, MA, USA), and DNA purification reagents were obtained from Qiagen (Hilden, Germany).

### 2.2 Bacterial strains and growth conditions

*Porphyromonas gingivalis* W50 and mutant strains were grown either on blood agar plates containing 5% defibrinated horse blood or in brain‐heart infusion broth (Oxoid, Basingstoke, UK) supplemented with hemin (5 μg/mL) in an anaerobic atmosphere consisting of 80% N_2_, 10% H_2_ and 10% CO_2_.^25^ Clindamycin HCl and tetracycline HCl were added to 5 μg/mL and 1 μg/mL respectively, for selection of *ermF* and *tet Q* in *P. gingivalis*. Ampicillin (Na^+^ salt; 100 μg/mL) or erythromycin (300 μg/mL) was added to the growth medium to select for pUC‐derived or *ermAM*‐containing plasmids respectively, in *Escherichia coli*.

### 2.3 Generation of *P. gingivalis* mutants

Purification and general manipulation of DNA, restriction mapping of plasmids and transformation of *E. coli* were as described previously.^25^, ^26^

A list of *P. gingivalis* strains used in this study is shown in the Table S1.

For the generation of *P. gingivalis* mutant strains *porR* and *galE*, chromosomal DNA from *P. gingivalis* W50 was used as the template for amplification/cloning purposes. The nomenclature originally used by TIGR is used throughout the manuscript. The genes encoding UDP‐glucose‐4‐epimerase *galE* and *porR* 23, 24, 27, 28 in *P. gingivalis* W50 were insertionally inactivated with *ermF‐ermAM* by allelic exchange following electro‐transformation and are described in detail in the Figure S1. The primers used in this study are listed in detail in Supplemental Methods.

### 2.4 Measurement of enzyme activity

Arg‐gingipain and Lys‐gingipain activities in whole cultures and culture supernatants of *P. gingivalis* and isogenic mutant strains were measured using *N*‐benzoyl‐dl‐arginine‐*p*‐nitroanilide (dl‐BR*p*NA) and *N*‐α‐acetyl‐l‐lysine‐*p*‐nitroanilide (l‐AcK*p*NA) respectively as substrates, in spectrophotometric assays, as previously described.^29^ Units of enzyme activity are expressed as change in absorbance at 405 nm/min per optical density at 600 nm (OD_600_) at 30°C. Enzyme activities were usually measured in triplicate using batches of bacterial cultures grown on different days. Student's *t* test for paired samples was used and the data were considered to be significant at a *P* value <.05.

### 2.5 SDS‐PAGE and SDS‐Urea‐PAGE

Sodium dodecyl sulfate (SDS)‐urea‐polyacrylamide gel electrophoresis (PAGE) of LPS was performed according to Inzana and Apicella.^30^ Samples were transferred onto nitrocellulose membranes and probed with MAb1B5, which recognizes the epitope Manα1‐2‐Manα1‐phosphate fragment in A‐PS of A‐LPS, as described previously.^27^ Silver staining of gels was performed using the Silver staining kit (Sigma‐Aldrich Co. Ltd.) according to the manufacturer's instructions.

### 2.6 Isolation of LPS and Lipid A

Lipopolysaccharide from *P. gingivalis* W50 and mutant strains for use in SDS‐urea‐PAGE experiments was prepared using an LPS extraction kit from Intron Biotechnology (South Korea).

Lipopolysaccharides used in hemin‐binding studies was prepared as described previously.^31^ De‐*O*‐phosphorylated LPS samples used in hemin‐binding experiments were prepared by dissolving LPS (10‐15 mg) in 0.5 mL of 48% aqueous hydrofluoric acid at 4°C and incubating at 4°C for 16 hours. Excess hydrofluoric acid was removed by dialysis against distilled water (6000‐8000 MWCO tubing) at 4°C followed by freeze‐drying.

De‐lipidation of LPS samples was carried out by treatment with 1.5% aqueous acetic acid at 100°C for 2‐4 hours in a heating block. Insoluble lipid A and traces of undegraded LPS were removed by ultracentrifugation at 30,000 *g* for 30 minutes at 10°C. The water‐soluble supernatant was lyophilized twice to remove all traces of acetic acid.

### 2.7 Hemin binding to whole cells of *P. gingivalis*

*Porphyromonas gingivalis* W50 and mutant strains were grown for 48 hours and cells were harvested by centrifugation (13 300 *g*) for 20 minutes at 4°C in Eppendorf tubes. The cells were washed with ice‐cold sterile PBS (3×1 mL) and stored at −70°C until required. Frozen cells were thawed and washed twice with 1 mL of sterile PBS. The cells were resuspended in PBS to give an OD_600_ of 1.25. Cell suspensions (0.8 mL) in triplicate were mixed with 0.2 mL of hemin solution containing 5 μg or 10 μg hemin and incubated at 37°C for 1 hour. Control samples contained 0.8 mL of PBS mixed with 0.2 mL of hemin solution containing 5 μg or 10 μg hemin (as above) for each set of experiments. The reaction mixture was centrifuged at 13,300 *g* for 20 minutes at 4°C, the supernatant was transferred to 1‐mL plastic disposable cuvettes and the OD_400_ was measured. Concentration of hemin in the supernatant was calculated from standard curves for hemin. The hemin bound (μg/OD_600_ of cells) was equal to the difference between the values for the control samples (hemin solution with no added cells, zero binding) and the supernatant from the experimental samples (bound hemin). The standard deviation was calculated.

For statistical analysis, a Student's *t* test for paired values was used, and data were considered to be significant at a *P* value <.05.

### 2.8 Binding of hemin to LPS

Freeze‐dried native LPS, de‐*O*‐phosphorylated LPS and de‐lipidated LPS samples from *P. gingivalis* W50 and *porR* were dissolved/resuspended in 0.05 m Tris‐HCl, pH 7.2 at a concentration of 1 mg/mL. Aliquots (50 μL) of LPS containing 50 μg was added to PBS (0.95 mL) containing 20 μg or 30 μg of hemin, in duplicate, in an Eppendorf tube and incubated at 37°C with shaking. LPS‐, de‐*O*‐phosphorylated LPS‐ and de‐lipidated‐LPS‐hemin complexes were pelletted by high speed centrifugation (30,000 *g*) for 60 minutes at 14°C.^32^ The amount of unbound hemin in the supernatant was determined by measuring the OD_400_ and the concentration was determined using a standard curve for hemin. The amount of hemin bound was calculated as the difference between the total hemin added to the reaction mixture and the amount present in the supernatant. The mean of two separate determinations±standard error of the mean was calculated ( http://www.upscale.utoronto.ca/PVB/Harrison/ErrorAnalysis/ ).

## 3 RESULTS

The *P. gingivalis* mutant strains used in this study have been described elsewhere (see Table S1). These include strains in which the genes encoding the proteases Rgps (*rgpA/rgpB*) and Kgp (*kgp*) have been inactivated, leading to loss of Arg‐gingipains and Lys‐gingipain, respectively,^33^ both of which have been strongly implicated in hemin acquisition by *P. gingivalis*.^34^

Inactivation of PG1051(*waaL*,O‐antigen ligase),^21^, ^31^ PG1142 (*wzy*, O‐antigen polymerase),^31^ and PG0129 (α‐1,3‐mannosyl transferase)^35^ lead to defects in LPS synthesis and have been described in detail elsewhere. In this manuscript, we have also studied *porR* where there is no A‐LPS synthesis and *galE*, which synthesizes O‐LPS, where the O‐PS repeating unit is shortened by a residue of Gal, in greater detail.

PorR is a putative transaminase and is homologous to the RfbE orthologue of *P. gingivalis* and belongs to the DegT Clusters of Orthologous Groups (COGs), the prototype of which is DegT of *Geobacillus* (*Bacillus*) *stearothermophilus*,^36^ which is involved in a range of biochemical functions including glycan synthesis, regulation of extracellular enzymes, altered control of sporulation, abnormal cell division, and loss of flagella.^36^ Proteins homologous to PorR have been found in several microorganisms involved in the biosynthesis of sugars present in capsular polysaccharide and aminoglycosides. In *P. gingivalis*, the inactivation of *porR* leads to pleiotropic effects involving loss of pigmentation, lack of synthesis of A‐LPS,^27^ processing of other proteins including fimbriae, and major alteration to the surface of the cell without perceptible effect on O‐LPS.^23^, ^24^, ^27^, ^28^ In addition, the Rgp isoforms, namely HRgpA and RgpB, which do not acquire the MAb1B5‐reactive glycan are present in the *porR* mutant strain whereas the isoforms that usually contain the MAb1B5 cross‐reactive epitope, namely RgpA_cat_ and mt‐Rgps^27^ are not synthesized. However, the synthesis of O‐LPS is not affected in the *porR* mutant strain and [^1^H]NMR spectroscopy of the O‐PS isolated from O‐LPS of this strain showed an identical [^1^H]NMR spectrum to that of O‐PS from the *P. gingivalis* W50 parent strain.^27^ Biologically, these effects translate to cell fragility, loss of recognition by antibodies of the periodontal patients’ sera, and an enhanced complement‐mediated killing as a result of the inability to synthesize A‐LPS.^23^, ^24^, ^27^, ^28^

### 3.1 Pigmentation and hemolysis of *P. gingivalis* strains

**Figure 1:**
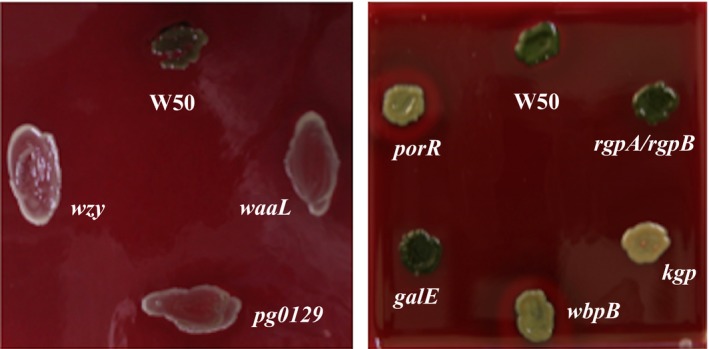
Pigmentation of *Porphyromonas gingivalis* strains on blood agar plates. *P. gingivalis* W50 and mutant strains were grown on blood agar plates for 7 d

*Porphyromonas gingivalis* W50, *rgpA/rgpB* and *galE* form brown‐ or black‐pigmented colonies on blood agar plates (Figure 1), whereas colonies of *kgp*,* porR*,* waaL*,* wzy*, and *pg0129* are non‐pigmented. Also shown is the *P. gingivalis* mutant strain *wbpB*, which has been described in detail elsewhere^28^, ^37^ and gives non‐pigmented colonies on blood agar plates.

### 3.2 Arg‐ and Lys‐gingipain activities in *P. gingivalis* strains

**Figure 2:**
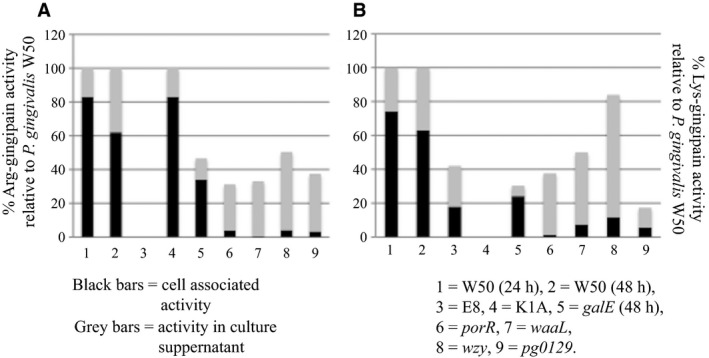
Arg‐gingipain and Lys‐gingipain activities in *Porphyromonas gingivalis* strains. *Porphyromonas gingivalis* W50 and isogenic mutant strains were grown in brain‐heart infusion broth for 24 or 48 h. Arg‐X (A) and Lys‐X (B) activities were measured using substrates dl‐BR *p* NA and L‐AcK*p* NA respectively as described in Methods. Enzyme activities are expressed as % activity relative to that of the parent *P. gingivalis* W50 strain (Absorbance_405 nm_ units/min/OD_600_). Black bars represent cell‐associated activities and grey bars represent enzyme activities in the culture supernatants

Arg‐gingipain and Lys‐gingipain activities in whole cultures and culture supernatants of *P. gingivalis* W50 and isogenic mutant strains were measured after either 24 or 48 hours and the results are shown in Figure 2A. The Arg‐gingipain (Rgp) activities present in the *P. gingivalis* strains vary widely. Total Rgp activity (100%) and activities present in cell‐associated and secreted forms in *P. gingivalis* W50 and *kgp* mutant strain are similar (~80% and ~20%, respectively) after 24 hours of growth, as expected. *Porphyromonas gingivalis rgpA/rgpB* mutant strain contains no Rgp activity, as expected. However, mutant strains *galE*,* porR*,* waaL*,* wzy*, and *pg0129* in which LPS synthesis is affected, contain lower levels of Rgp activity compared with the parent W50 strain (*P* values <.0003). In addition, in mutant strains *porR*,* waaL*,* wzy*, and *pg0129*, almost all the enzyme activity (~90%‐100%) is secreted into the supernatant after 24 hours of growth compared with the parent W50 strain, in which only ~20% of Rgp activity is shed into the supernatant. Although *galE* contains ~50% of total Rgp activity compared with the W50 parent strain after 48 hours of growth, the distribution of enzyme activity between cell‐associated and secreted forms was similar to that of the parent W50 strain: ~30% and ~20% of Rgp activity in cell‐associated and supernatant forms in *galE* compared with ~60% and ~40% of Rgp activity in cell‐associated and supernatant forms in *P. gingivalis* W50.

Similarly, the Lys‐gingipain (Kgp) activities (Figure 2B) in whole cultures of the *P. gingivalis* mutant strains also show wide variation. *P. gingivalis* W50 contains the highest amount of Kgp activity. As expected, *kgp* shows no detectable Kgp activity. The amounts of Kgp activity in cell‐associated and culture supernatants also show wide variation (Figure 2B). However, in *P. gingivalis* mutant strains, namely *porR*,* waaL*,* wzy*, and *pg0129*, almost all the Kgp activity is present in the culture supernatant after 24 hours of growth, which is very similar to that observed for Rgp activity. Although the Rgp and Kgp activities of the *P. gingivalis* mutant strains show great variation, these results highlight the properties of the isogenic mutant strains *porR*,* waaL*,* wzy*, and *pg0129*, where almost all the Rgp and Kgp activities are released into the culture supernatant after 24‐48 hours of growth indicating the lack of tethering/anchoring molecules on the cell surface of these strains, which would otherwise enable these enzymes from being shed into the culture medium. As the mutant strains *porR*,* waaL*,* wzy*, and *pg0129* are defective in LPS biosynthesis, the inability to retain the gingipains on the cell surface could be a direct result of this deficiency.

### 3.3 Cross‐streaking experiments

**Figure 3:**
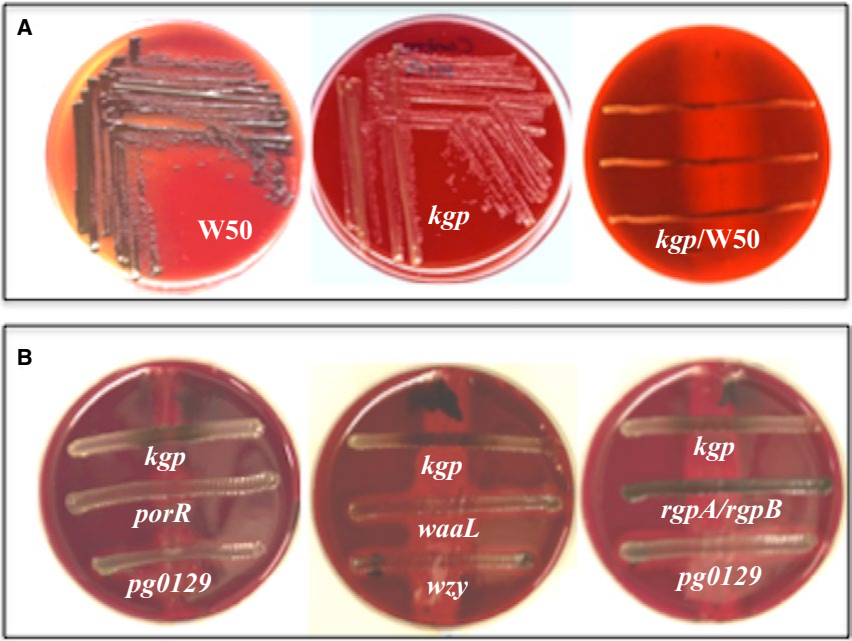
Cross‐streaking of *Porphyromonas gingivalis kgp* mutant strain on blood agar. (A) *P. gingivalis* W50 on blood agar, *kgp* on blood agar, *P. gingivalis* W50 was initially streaked on a blood agar plate and following the formation of a zone of hemolysis (3 d), the cells were removed with a swab containing clindamycin to suppress regrowth of the wild‐type strain and the plates were cross‐streaked with *kgp*. Note pigmentation of the *kgp* mutant cells takes place only on the zone of hemolysis produced by the parent strain. (B) Blood agar plates were initially streaked with W50 as in (A). Plates were cross‐streaked with r*gpA/rgpB*,* kgp*,* porR*,* pg0129*,* waaL*, and *wzy*. Pigmentation of *kgp* takes place on the zone of hemolysis whereas the other strains do not pigment even after 6 d of growth

*Porphyromonas gingivalis* W50 was initially streaked on a blood agar plate and following the formation of a zone of hemolysis (3 days), the cells were removed with a swab containing clindamycin to suppress regrowth of the wild‐type strain and the plates were cross‐streaked with *P. gingivalis* mutant strains (Figure 3). Although *rgpA/rgpB* and *kgp* give brown and non‐pigmenting colonies when grown on blood agar plates because of the lack of Rgps and Kgp, respectively, they do pigment when cross‐streaked on plates on which *P. gingivalis* W50 has been previously grown and caused hemolysis (Figure 3). This suggests that *rgpA/rgpB* and *kgp* have the ability to pigment if supplied with externally added hemin. However, cross‐streaking of *P. gingivalis porR*,* waaL*,* wzy*, and *pg0129* strains on blood agar plates as above did not cause the deposition of hemin/black pigment on the surfaces of these cells (Figure 3). This indicates that the mutant strains are unable to harness any available hemin in the environment and retain it on their cell surface.

### 3.4 Analysis of LPS

**Figure 4:**
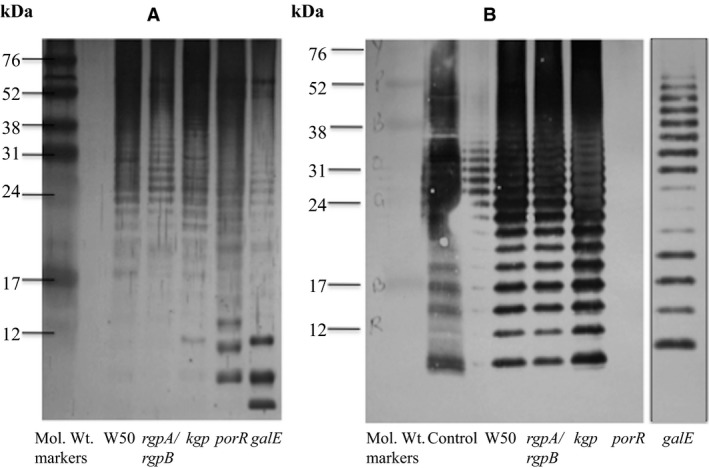
Sodium dodecyl sulfate‐urea‐PAGE and silver staining of lipopolysaccharide (LPS) from *Porphyromonas gingivalis* W50 and mutant strains (A). Western blotting vs MAb 1B5 of LPS from *P. gingivalis* W50 and mutant strains (B). LPS purified as described in Methods were subjected to SDS‐urea‐PAGE. The *P. gingivalis* strains used in the isolation of LPS are indicated below the lanes. The control sample in (B) is the phenol extract of *P. gingivalis* W50 cells containing predominantly LPS

The SDS‐urea‐PAGE followed by silver staining of LPS purified from *P. gingivalis* W50 and mutant strains *rgpA/rgpB*,* kgp*,* porR*, and *galE* show the characteristic laddering pattern (Figure 4A). However, in *porR* and *galE*, the O‐LPS shows a higher intensity of bands in the core‐, core‐plus one repeating unit and core‐plus two repeating units (Figure 4A). In the *P. gingivalis galE* mutant strain, the O‐PS repeating unit, [→3)‐α‐D‐Gal*p*‐(1→6)‐α‐D‐Glc*p*‐(1→4)‐α‐L‐Rha*p*‐(1→3)‐β‐D‐GalNAc*p*‐(1→] is shortened by one Gal residue (unpublished data). The SDS‐urea‐PAGE of LPS from *P. gingivalis waaL*,* wzy*, and *pg0129* mutant strains have been described elsewhere^21^, ^31^, ^35^ and are not shown here.

Sodium dodecyl sulfate‐urea‐PAGE of LPS from *P. gingivalis* W50 and mutant strains followed by silver staining indicate that all these strains synthesize O‐LPS. SDS‐urea‐PAGE of LPS followed by Western blotting vs MAb 1B5, which recognizes the epitope Manα1‐2‐Manα1‐phosphate fragment in A‐PS of A‐LPS^27^ show that W50, *rgpA/rgpB*,* kgp*, and *galE* also synthesize A‐LPS (Figure 4B) as indicated by the laddering pattern and immunoreactivity with MAb 1B5. However, *porR* synthesizes only O‐LPS and A‐LPS is absent as shown by the lack of cross‐reactivity with MAb 1B5 (Figure 4B).

### 3.5 Hemin binding

#### 3.5.1 Hemin binding by whole cells

**Figure 5:**
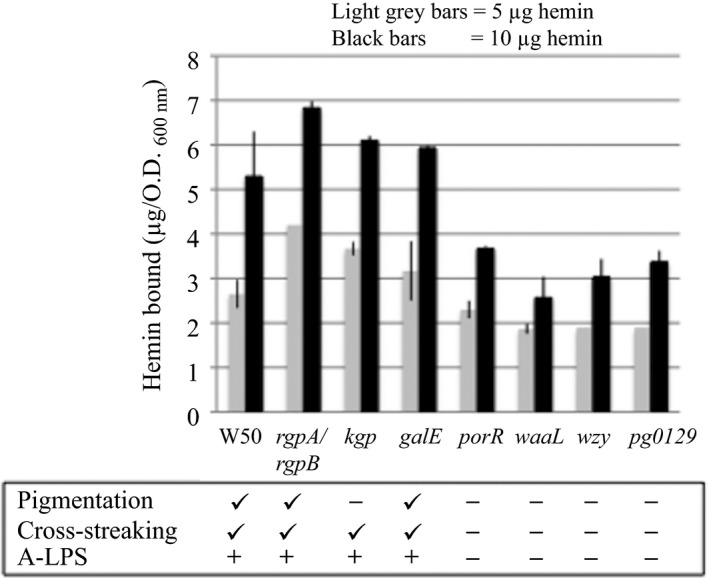
Hemin binding by whole cells of *Porphyromonas gingivalis* W50 and isogenic mutant strains. The *P. gingivalis* W50 and isogenic mutant strains were grown in brain‐heart infusion broth for 48 h. Details of hemin binding are as described in Methods. The amount of hemin bound by the cells (μg hemin bound/OD_600_ cells) at the added concentrations of hemin of 5 μg and 10 μg are shown. The characteristics of the *P. gingivalis* strains are indicated below the figure. Statistical analyses (*P* values by Student's *t* test) of the amount of hemin bound by W50 and *P. gingivalis* mutant strains are also indicated. *P*‐values for hemin‐binding by strains *rgpA/rgpB*,* kgp* and *galE* compared with W50 were >.05 whereas *P*‐values for hemin‐binding by strains *porR*,* waaL*,* wzy*, and *pg0129* compared with W50 were <.0003

Hemin binding by whole cells of *P. gingivalis* W50, *rgpA/rgpB*,* kgp*,* galE*,* porR*,* waaL*,* wzy*, and *pg0129* was measured as described in the Methods section and the results obtained are shown in Figure 5. *Porphyromonas gingivalis* W50 and mutant strains *rgpA/rgpB* and *galE*, which pigmented brown and black on blood agar plates, respectively (Figure 1) showed hemin binding values (5.6 μg/OD_600_, 6.8 μg/OD_600_ and 5.9 μg/OD_600_, respectively), at the highest concentration (10 μg/mL) of hemin used in the binding experiments. Although *kgp* was non‐pigmenting on blood agar plates due to the absence of Kgp, it shows hemin binding (6.1 μg/OD_600_) when supplied with hemin (also observed when *kgp* is cross‐streaked on blood agar plates on which *P. gingivalis* W50 was previously grown (Figure 3). The *P. gingivalis* mutant strains *porR*,* waaL*,* wzy*, and *pg0129*, which were non‐pigmenting on blood agar plates, were able to bind between ~2.5 and 3.7 μg of hemin/OD_600_ of cells, which is ~45%‐65% of hemin bound by the parent W50 strain. Hence, *P. gingivalis* mutant strains that do not synthesize A‐LPS show reduced hemin binding.

#### 3.5.2 Hemin binding by LPS

**Figure 6:**
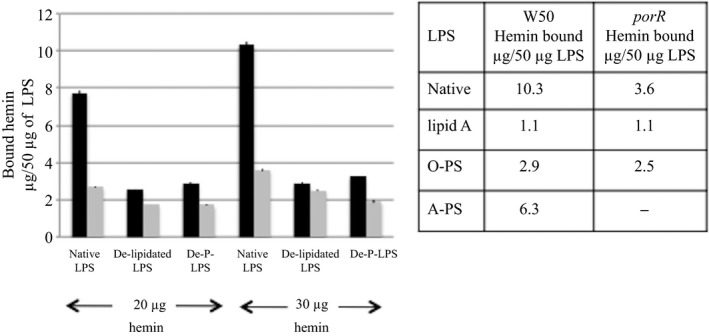
Hemin binding to lipopolysaccharide (LPS), de‐lipidated LPS and de‐*O*‐phosphorylated LPS derived from *Porphyromonas gingivalis* strains. The hemin‐binding studies were performed as described in the Methods. Black bars=LPS and derivatives from *P. gingivalis* W50 and grey bars=LPS and derivatives from *porR* mutant strain. Inset: hemin binding to native LPS, lipid A, O‐PS and A‐PS derived from *P. gingivalis* and *porR* mutant strains

Hemin binding to LPS isolated from *P. gingivalis* W50 and mutant strain *porR* grown in brain‐heart infusion were measured at two different concentrations of hemin, namely 20 μg/mL and 30 μg/mL and are shown in Figure 6. At 30 μg/mL of added hemin, there is a slightly higher amount of hemin bound by all the LPS (Figure 6) compared with the amounts bound at 20 μg/mL of hemin. Henceforth, all the values for hemin binding to LPS will only refer to those obtained at the higher concentration of hemin used in the experiment, namely 30 μg/mL. LPS from *P. gingivalis* W50 is able to bind hemin at ~10.3 μg/50 μg of LPS. However, LPS from *porR*, which is devoid of A‐LPS^27^ was able to bind 3.6 μg hemin/50 μg of LPS, which is considerably lower than that of LPS from the parent W50 strain.

To determine the extent of hemin binding to the lipid A portion of the LPS molecule, the LPS was delipidated and the binding of hemin to the resulting polysaccharide (PS) was measured. In addition to removal of lipid A, delipidation of LPS causes the destruction of A‐PS^27^ whereas O‐PS is largely unaffected.^20^ The binding of hemin by de‐lipidated LPS (Figure 6) from *P. gingivalis* W50 is reduced to ~2.9 μg/50 μg of LPS and hemin binding by de‐lipidated *porR* LPS is reduced to 2.5 μg hemin/50 μg of LPS. These results enable us to apportion the extent of hemin binding by A‐LPS, O‐LPS and lipid A. As *porR* LPS contains only O‐LPS, the hemin bound (~3.6 μg/50 μg of LPS) must be due to O‐LPS. De‐lipidation of *porR* LPS reduces the hemin bound to ~2.5 μg/50 μg of LPS suggesting that ~1.1 μg of hemin bound to de‐lipidated LPS must be due to binding to lipid A and the remaining ~2.5 μg of hemin bound to 50 μg of LPS must be due to binding to the O‐PS component of O‐LPS. The hemin binding to de‐lipidated LPS from *P. gingivalis* W50 and *porR* strains is remarkably similar (2.9 μg vs 2.5 μg hemin bound to 50 μg of de‐lipidated LPS, respectively) whereas hemin bound to native LPS differs greatly, 10.3 μg vs 3.6 μg hemin bound to W50 and *porR* LPS respectively. Hence, it can be deduced that ~6.3 μg of hemin bound/50 μg of native LPS in *P. gingivalis* W50 must be due to binding to A‐LPS.

We also investigated the binding of hemin to de‐*O*‐phosphorylated LPS derived from the parent W50 and *porR* mutant strain. De‐*O*‐phosphorylation of O‐LPS and A‐LPS does not cause de‐polymerization of the PS chains, but results in loss of phosphoethanolamine in O‐PS^20^ and in the loss of the cross‐reacting epitope Manα1‐2Manα1‐phosphate in A‐LPS.^21^, ^27^ The results (Figure 6) show that the binding of hemin is reduced in de‐*O*‐phosphorylated LPS from the parent and *porR* mutant strain to ~3.3 μg and 1.9 μg, respectively. Hence, the reduction in binding of hemin caused by de‐*O*‐phosphorylation of LPS from *P. gingivalis* W50 compared with that for LPS from *porR* mutant strain suggests that negatively‐charged A‐LPS may have an important role to play in the binding/deposition of hemin on the cell surface of *P. gingivalis*.

## 4 DISCUSSION

The hemin binding properties of *P. gingivalis* have been a major area of study and interest for several years. Iron utilization systems in *P. gingivalis* are quite complex and several proteins have been implicated in hemin release from the host, to its transport and deposition on the bacterial cell surface. Virulence of *P. gingivalis* is closely associated with the ability of the organism to pigment, namely to the deposition of the μ‐oxo‐bisheme on the cell surface. The requirement for the dimeric Arg‐gingipain (Rgp), HRgpA, and Lys‐gingipain (Kgp) in the release of heme groups from hemoglobin and the formation of the μ‐oxo‐bisheme complex is very well characterized.^38^ However, the cell‐surface molecules required for retention of μ‐oxo‐bisheme and pigmentation have not been fully elucidated.

Haem‐starved *P. gingivalis* ATCC33277 and WT40 expressed two binding sites for hemin, a low‐abundance high‐affinity site (1000‐1500 sites/cell) of *K*_d_ between 3.6×10^−11^ and 9.6×10^−11^ m and a high‐abundance low‐affinity site (1.9×10^5^ to 6.3×10^5^ sites/cell) where the estimated *K*_d_ ranged between 2.6×10^−7^ and 6.5×10^−8^ m.^22^ Treatment with *N*‐bromosuccinimide inactivated hemin binding by both sites in *P. gingivalis*, whereas pronase treatment caused only a limited reduction in hemin binding probably because only one of the two sites was sensitive to pronase. Tompkins et al.^22^ concluded that the higher‐affinity site was probably exposed on the surface of *P. gingivalis* and sensitive to pronase whereas the lower‐affinity site may be sequestered within the outer membrane, especially if it functioned to store hemin.

The black heme‐pigment deposited on the cell surface of *P. gingivalis* and which serves as an iron source for this organism, is composed of μ‐oxo‐bisheme [Fe(III)PPIX]_2_O, and the multidomain cysteine proteases Arg‐gingipains and Lys‐gingipain acting in concert have been shown to be important in the production of μ‐oxo‐bisheme from oxyhemoglobin.^34^ HRgpA, the dimeric isoform of RgpA, promotes the formation of methemoglobin from oxyhemoglobin, which is degraded by Kgp to form the black pigment μ‐oxo‐bisheme.^10^ Hence, both Arg and Lys gingipains are required for the production of the black pigment in *P. gingivalis*.

In the absence of Rgps, no μ‐oxo‐bisheme is produced, although the double knockout *P. gingivalis rgpA/rgpB* strain, which lacks Rgps, gave brown‐colored colonies even after prolonged incubation on blood agar plates. The brown pigment contained an Fe(III) hemoglobin‐hemichrome complex as the major heme‐containing species.^38^ The heme from the complex was transferred to albumin after prolonged incubation of cells with oxyhemoglobin in the presence of albumin and this was tightly bound to the cell surface in the *P. gingivalis* (*rgpA/rgpB*) strain.

*Porphyromonas gingivalis* W50 does not pigment when grown in liquid broth cultures with added hemin (5 mg/L), but gives black‐pigmented colonies when grown on blood agar plates due to the deposition of μ‐oxo‐bisheme, derived from hemoglobin, on the cell surface. This difference may suggest that the source of hemin is critical for the pigmentation process. Here, we have shown that *P. gingivalis* mutants *rgpA/rgpB* and *kgp* do not normally pigment, but produce black‐pigmented colonies when cross‐streaked on plates on which *P. gingivalis* W50 was previously grown and caused hemolysis. These observations show that the ability to retain the pigment on the *P. gingivalis* cell surface can be uncoupled from the ability to release heme from hemoglobin (with the concomitant formation of μ‐oxo‐bisheme) by the combined action of Rgps and Kgp. This behavior mirrors the ability of the cells of *rgpA*/*rgpB* and *kgp* mutant strains to bind externally added hemin to the same extent as the parent W50 strain when hemin‐binding was measured in liquid suspensions (Figure 5). Therefore, we propose that the ability of the bacterial cells to bind hemin may parallel the retention of μ‐oxo‐bisheme on the cell surface when the strains are grown on blood agar plates. The inability of *P. gingivalis* mutant strains *porR*,* waaL*,* wzy*, and *pg0129* to produce black‐pigmented colonies in cross‐streaking experiments is supported by the reduced binding of hemin to cells of these strains. The major difference between the *P. gingivalis* mutant strains, which have the ability to acquire μ‐oxo‐bisheme (*rgpA*/*rgpB* and *kgp*) on cross‐streaking and those mutant strains that lack this property (*porR*,* waaL*,* wzy*, and *pg0129*) is the production of A‐LPS by pigmenting strains.

**Figure 7:**
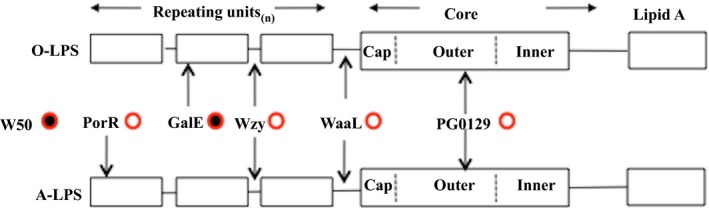
Structure of *Porphyromonas gingivalis* lipopolysaccharide (LPS) and role of A‐LPS in pigmentation. The *P. gingivalis* W50 parent strain synthesizes two LPS: O‐LPS and A‐LPS and is black‐pigmented on blood agar (filled circle). Inactivation of Wzy (O‐antigen polymerase) leads to a core‐plus‐one repeating unit structure for both LPS. Inactivation of either WaaL (O‐antigen ligase) or PG0129 (alpha‐1,3‐mannosyl‐transferase) leads to the absence of A‐PS and O‐PS. In all three cases, the mutants lose the ability to pigment (open circles). Inactivation of GalE affects the synthesis of the O‐PS but not A‐PS and pigmentation is unaffected. Inactivation of PorR abolishes the synthesis of A‐LPS but not O‐LPS and this mutant fails to pigment

This suggests that the *P. gingivalis* cell surface must contain a molecule that provides a scaffold/matrix for the deposition and retention of any hemin or pigment that is produced/acquired by the organism. Figure 7 shows a simplified diagram of the pigmentation characteristics and the types of LPS synthesized by the *P. gingivalis* strains used in this study.

Transmission electron microscopy of *P. gingivalis porR* 27, 28 and *waaL* mutant strains (which lack A‐LPS)^21^ show that their extracellular surface layers are of reduced thickness compared with the W50 parent and *rgpA/rgpB* mutant strains (which do synthesize A‐LPS) and the cells appear more fragile based on the rate of decrease of the culture OD in the stationary phase.^21^, ^23^, ^27^ Shoji et al.^23^ suggested that strains that were unable to synthesize A‐LPS probably lack a tethering/anchoring molecule(s) on their cell surface, which retain gingipains and this could explain the release of Arg‐ and Lys‐gingipains into the culture supernatants in *P. gingivalis porR*,* waaL*,* wzy*, and *pg0129* mutant strains, which are defective in the LPS‐biosynthetic pathway.

Grenier reported that the lipid A component of LPS mediated the binding of uncomplexed hemin by *P. gingivalis*.^39^ As hemin is a lipophilic molecule, it would be expected to bind to Lipid A/LPS. *Escherichia coli*, which does not require exogenous heme when grown in iron‐replete conditions, was shown to bind as much uncomplexed hemin as *Prevotella intermedia*. This effect was inhibited by albumin, which indicated that when heme is provided in the free form, most of it binds to the bacterium with an affinity lower than that for albumin. However, Tompkins et al.^22^ concluded that most gram‐negative bacteria would exhibit similar non‐specific hemin binding and that the LPS‐mediated hemin binding is probably not biologically relevant because of the low affinity of the interaction and the presence of large amounts of host plasma proteins, which function to counter the lipophilic disposition of hemin. They showed that treatment of *P. gingivalis* cells with pronase caused a slight reduction in, but did not eliminate, hemin‐binding and the authors suggested that this was probably due to the pronase‐sensitive hemin‐binding sites not being exposed on the surface of the cell and therefore not digested by pronase treatment.^22^ However, it seems more plausible that it is the presence of A‐LPS (which is not sensitive to pronase treatment) on the surface of *P. gingivalis* that acts as a site for the deposition/binding of hemin.

Studies on hemin binding to whole cells of *P. gingivalis* W50 and mutant strains and hemin binding to native LPS and de‐lipidated LPS from *P. gingivalis* W50 and *porR* strains show that absence of A‐LPS causes a reduction in hemin binding. Hence, absence of A‐LPS in the extracellular surface of *P. gingivalis* eliminates or reduces a scaffold/anchoring mechanism not only for retention of Arg‐ and Lys‐gingipains but also for the deposition of μ‐oxo‐bisheme pigment or hemin derived from the environment and highlights the importance of A‐LPS in the virulence of this organism.
