# Supplementary figures and images for: Hemin binding by Porphyromonas gingivalis strains is dependent on the presence of A‐LPS
Source: Mol Oral Microbiol. 2017 Mar 9;32(5):365–74. doi: 10.1111/omi.12178 (PMC5600137; doi:10.1111/omi.12178)

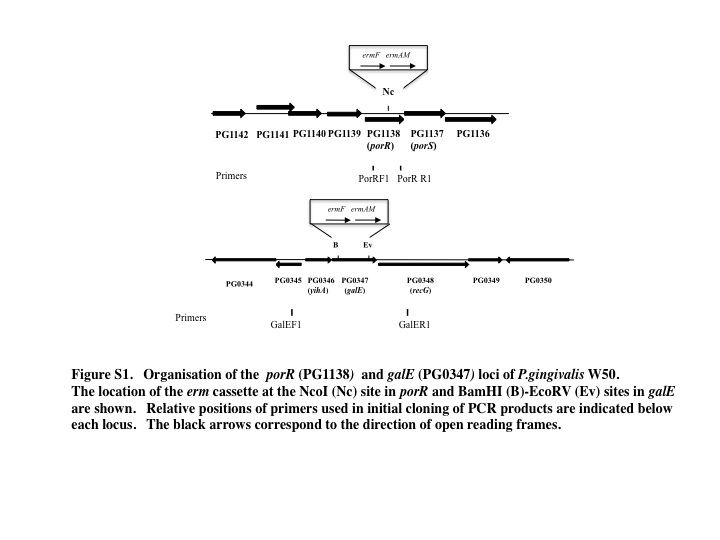

Supplement: Supplementary file 1 [file OMI-32-365-s001.tif]
