# Supplementary material for: Hemin binding by Porphyromonas gingivalis strains is dependent on the presence of A‐LPS
Source: Mol Oral Microbiol. 2017 Mar 9;32(5):365–74. doi: 10.1111/omi.12178 (PMC5600137; doi:10.1111/omi.12178)
Supplement: Supplementary file 2 [file OMI-32-365-s002.docx]

**Table S1. List of strains used in this study.**

| *Porphyromonas gingivalis* strain | Common name- assigned function | Genes^a^ | Genotype^b^ | Reference |
| --- | --- | --- | --- | --- |
| W50 |  |  | Wild type parent | Curtis et al. (1991) |
| E8 (*rgpA/rgpB*) | Arg-gingipains A & B | PG0506/  PG2024 | *rgpA*:: *tetQ*; *rgpB*:: *erm* | Aduse-Opoku et al. (2000); Curtis et al. (2002) |
| K1A (*kgp*) | Lys-gingipain | PG1844 | *kgp*::*erm* | Aduse-Opoku et al. (2000); Curtis et al. (2002) |
| *porR* | PorR | PG1138 | PG1138::*erm* | Gallagher et al. (2003); Paramonov et al. (2005); Slaney et al. (2006) |
| PG1051 (*waaL*) | Oligosaccharyl transferase | PG1051 | PG1051::*erm* | Rangarajan et al. (2008) |
| PG1142 (*wzy*) | Wzy, O-antigen polymerase | PG1142 | PG1142::*erm* | Paramonov et al. (2009) |
| PG0129 | Mannosyl transferase | PG0129 | PG0129::*erm* | Paramonov et al. (2015) |
| *galE* | UDP-Glc-UDP-Gal epimerase | PG0347 | PG0347::*erm* | This paper |
| *wbpB* | WbpB | PG2119 | PG2119::*erm* | Slaney et al. (2006); Shoji et al. (2014). |

Notes:

^a^ Gene loci names are based on the genome of *Porphyromonas gingivalis* W83 (Nelson et al. (2003). ^b^ *erm* cassette is a composite of *ermF-ermAM*.
