# Supplementary material for: Hemin binding by Porphyromonas gingivalis strains is dependent on the presence of A‐LPS
Source: Mol Oral Microbiol. 2017 Mar 9;32(5):365–74. doi: 10.1111/omi.12178 (PMC5600137; doi:10.1111/omi.12178)
Supplement: Supplementary file 3 [file OMI-32-365-s003.docx]

**SUPPLEMENTAL.**

**METHODS.**

Generation of *P. gingivalis* mutant strains *porR* and *galE*.

Chromosomal DNA from *P.gingivalis* W50 was used as the template for amplification/ cloning purposes. The nomenclature originally used by TIGR is used throughout. The genes encoding UDP-Glucose-4-epimerase *galE* (PG0347) and *porR* (PG1138 (Shoji et al., 2002; Gallagher et al., 2003; Paramonov et al., 2005; Slaney et al., 2006) in *P.gingivalis* W50 were insertionally inactivated with *ermF-ermAM* by allelic exchange following electro-transformation.

Primer pairs incorporating NotI sites (in bold), PorRF1: *atatat****gcggccgc***TTGCGGAAGATTTGGCAG and PorRR1: *atatat****gcggccgc***GGGATGGAGAGAACAGTTCG were used to amplify *porR*(PG1138).

GalEF1: *atatat****gcggccgc***GGCATCAACGATCCATACG and GalER1: *atata****tgcggccgc***GAGTACGTACAGGAGTTGCTGG were used to amplify *galE* in PCR master mix (Extensor mix Reddy Load PCR master mix (Buffer 2, Thermoscientific)) as previously described (Aduse-Opoku et al., 2006). The amplicons were cloned at the NotI site of pUC18not and inserts of *porR* (937 bp) and *galE* (2545 bp) were further manipulated to ligate a 2.1 kb *erm* cassette (Fletcher et al., 1995) at the unique NcoI and BamHI-EcoRV sites respectively. NotI-restricted plasmids were used to electrotransform 6h grown cells of *P.gingivalis* W50 to clindamycin resistance. PCR was performed on purified chromosomal DNA from six separate isolates of each mutant strain. The original primers were used to amplify the region *porR*::*erm* and *galE*::*erm* to show correct insertion of the *erm* cassette. One strain from each was chosen and designated as either *porR* or *galE* (Fig. S1).

Gallagher et al. (2003) have referred to a *porR* mutant strain isolated by inactivation of PG1138 in *P. gingivalis* W50 which is described in greater detail in this manuscript.

**Description of PorR.**

PorR is a putative transaminase and is homologous to RfbE orthologue of *P. gingivalis* and belongs to the DegT Clusters of Orthologous Groups (COGs), the prototype of which is DegT of *Geobacillus* (*Bacillus*) *stearothermophilus* (Takagi et al., 1990) which is involved in a range of biochemical functions including glycan synthesis, regulation of extracellular enzymes, altered control of sporulation, abnormal cell division and loss of flagella (Takagi et al., 1990). Proteins homologous to PorR have been found in several microorganisms involved in the biosynthesis of sugars present in capsular polysaccharide and aminoglycosides. In *Vibrio cholerae* O1 and *E. coli* O157, *rfbE* encodes perosamine synthetase (Bilge et al., 1996; Albermann & Piepersburg, 2001) and the *rfbE* orthologue *per* in *Caulobacter crescentus* also encodes a perosamine synthetase (Awram & Smit, 2001). In *P. gingivalis*, the inactivation of *porR* leads to pleiotropic effects involving pigmentation, lack of synthesis of A-LPS (Paramonov et al., 2005), processing of other proteins including fimbriae, and major alteration to the surface of the cell without perceptible effect on O-LPS (Shoji et al., 2002; Gallagher et al., 2003; Paramonov et al., 2005; Slaney et al., 2006). In addition, the Rgp isoforms namely HRgpA and RgpB which do not acquire the MAb1B5 reactive glycan are present in the *porR* mutant strain whereas the isoforms which usually contain the MAb 1B5 cross-reactive epitope, namely RgpA_cat_ and mt-Rgps (Paramonov et al., 2005) are not synthesised. However, the synthesis of O-LPS is not affected in the *porR* mutant strain and ^1^H-NMR spectroscopy of the O-PS isolated from O-LPS of this strain showed an identical ^1^H-NMR spectrum to that of O-PS from the *P. gingivalis* W50 parent strain (Paramonov et al., 2005). Biologically, these effects translate to cell fragility, loss of recognition by antibodies of the periodontal patients’ sera, and an enhanced complement mediated killing as a result of the inability to synthesise A-LPS (Gallagher et al., 2003; Shoji et al., 2002; Paramonov et al., 2005; Slaney et al., 2006).

REFERENCES.

- Aduse-Opoku, J., Davies, N.N., Gallagher, A. et al. (2000) Generation of Lys-gingipain protease activity in *Porphyromonas gingivalis* W50 is independent of Arg-gingipain protease activities. *Microbiology* **146**: 1933-1940.

Aduse-Opoku, J., Slaney, J.M., Hashim, A. et al. (2006) Identification and characterization of the capsular polysaccharide (K-antigen) locus of *Porphyromonas gingivalis*. *Infect Immun* **74**: 449-460.

Albermann, C., and Piepersberg, W. (2001) Expression and identification of the RfbE protein from *Vibrio cholerae* O1 and its use for the enzymatic synthesis of GDP-D-perosamine. *Glycobiology* **11**: 655-661.

Awram, P., and Smit, J. (2001) Identification of lipopolysaccharide O antigen synthesis genes required for attachment of the S-layer of *Caulobacter crescentus*. *Microbiology* **147**: 1451-1460.

Bilge, S.S., Vary, J.C., Jr., Dowell, S.F. and Tarr, P.I. (1996) Role of the *Escherichia coli* O157:H7 O side chain in adherence and analysis of an *rfb* locus. *Infect Immun* **6**4: 4795-4801.

Curtis, M.A., Slaney, J.M., Carman, R.J. and Johnson, N.W. (1991) Identification of the major surface protein antigens of *Porphyromonas gingivalis* using IgG antibody reactivity of periodontal case-control serum. *Oral Microbiol Immunol* **6**: 321-326.

Curtis, M.A., Aduse Opoku, J., Rangarajan, M. et al. (2002) Attenuation of the virulence of *Porphyromonas gingivalis* by using a specific synthetic Kgp protease inhibitor. *Infect Immun* **70**: 6968-6975.

Fletcher, H.M., Schenkein, H.A., Morgan, R.M., Bailey, K.A., Berry, C.R. and Macrina, F.L. (1995) Virulence of a *Porphyromonas gingivalis* W83 mutant defective in the *prtH* gene. *Infect Immun* **63**: 1521-1528.

- Gallagher, A., Aduse-Opoku, J., Rangarajan, M., Slaney, J.M. and Curtis, M.A. (2003) Glycosylation of the Arg-gingipains of *Porphyromonas gingivalis* and comparison with glycoconjugate structure and synthesis in other bacteria. *Current Protein and Peptide Science* **4**: 427-441.

Nelson, K.E., Fleischmann, R.D., DeBoy, R.T. et al. (2003) Complete genome sequence of the oral pathogenic Bacterium *Porphyromonas gingivalis* strain W83. *J Bacteriol* **185**: 5591-5601.

Paramonov, N., Rangarajan, M., Hashim, A. et al. (2005) Structural analysis of a novel anionic polysaccharide from *Porphyromonas gingivalis* strain W50 related to Arg-gingipain glycans. *Mol Microbiol* **58**: 847-863.

Paramonov, N., Aduse-Opoku, J., Hashim, A., Rangarajan, M., and Curtis, M.A. (2009) Structural Analysis of the Core Region of O-Lipopolysaccharide of *Porphyromonas gingivalis* from Mutants Defective in O-Antigen Ligase and O-Antigen Polymerase. *J Bacteriol* **191**: 5272-5282.

- Paramonov, N., Aduse-Opoku, J., Hashim, A., Rangarajan, M., and Curtis, M.A. (2015) Identification of the Linkage between A-Polysaccharide and the Core in the A-Lipopolysaccharide of *Porphyromonas gingivalis* W50. *J Bacteriol* **197**: 1735-1746.

Rangarajan, M., Aduse-Opoku, J., Paramonov, N. et al. (2008) Identification of a Second Lipopolysaccharide in *Porphyromonas gingivalis* W50. *J Bacteriol* **190**: 2920-2932.

Shoji, M., Ratnayake, D.B., Shi, Y. et al. (2002) Construction and characterization of a non-pigmented mutant of *Porphyromoinas gingivalis*: cell surface polysaccharide as an anchorage for gingipains. *Microbiology* **148**: 1183-1191.

Shoji, M., Sato, K., Yukitake, H., Naito, M. and Nakayama, K. (2014) Involvement of the Wbp pathway in the biosynthesis of *Porphyromonas gingivalis* lipopolysaccharide with anionic polysaccharide. *Scientific Reports* **4**: 5056-5064.

- Slaney, J.M., Gallagher A., Aduse-Opoku, J., Pell, K. and Curtis, M.A. (2006) Mechanisms of resistance of *Porphyromonas gingivalis* to killing by serum complement. *Infect Immun* **74**: 5352-5361.
- Takagi,M., Takada,H., and Imanaka,T. (1990) Nucleotide sequence and cloning in *Bacillus subtilis* of the *Bacillus stearothermophilus* pleiotropic regulatory gene *degT*. *J Bacteriol* **172**: 411-418.
- Table S1. List of Strains used in this study.**Figure S1.**

Organisation of the *porR* (PG1138) and *galE* (PG0347) loci in *P. gingivalis* W50*.*

The location of the *erm* cassette at the NcoI (Nc) site in *porR* and BamHI (B)-EcoRV (Ev) sites in *galE* are shown*.* Relative positions of primers used in initial cloning of PCR products are indicated below each locus. The black arrows correspond to the directions of open reading frames.
